# Measurement of longitudinal tibial nerve excursion during ankle joint dorsiflexion: an in-vivo investigation with ultrasound imaging

**DOI:** 10.1186/1757-1146-5-S1-O37

**Published:** 2012-04-10

**Authors:** Matthew Carroll, Janet Yau, Keith Rome, Wayne Hing

**Affiliations:** 1 Health and Rehabilitation Research Institute, AUT University, Auckland, 0627, New Zealand; 2 Department of Podiatry, AUT University, Auckland, 0627, New Zealand; 3 Department of Physiotherapy, AUT University, Auckland, 0627, New Zealand

## Background

A key mechanical function of peripheral nerves is their ability to slide in relation to the surrounding tissues. This function is of paramount importance to maintain ideal neural function [1]. Advances in ultrasound imaging and the development of specific software (cross-correlation analysis) have made it possible to analyse real-time ultrasound images, allowing for quantification of *in-vivo* peripheral nerve movement [2]. Cross-correlation analysis has been utilised in numerous upper extremity *in-vivo* neural investigations [3-5]. No study has investigated *in-vivo* longitudinal nerve excursion at the ankle joint. The aims of this study were to quantify the degree of longitudinal tibial nerve excursion as the ankle moved from dorsiflexion to plantarflexion and assess the between session intra-rater reliability of the ultrasound imaging technique.

## Materials and methods

A sample of sixteen participants (10 male, 6 female; mean [SD] age 34.7 [9.3] years old) were recruited. A three second video loop of the tibial nerve was captured by ultrasound imaging as the ankle moved from 20° plantarflexion to 10° dorsiflexion. The tibial nerve was imaged on two occasions with a 5 minute interval between measurement sessions. Foot and ankle position was standardised on a measurement platform. Video loops were analysed to determine the degree of longitudinal nerve excursion. Intraclass correlation coefficients (ICC), with 95% confidence intervals (CI), standard error of the measurement (SEM) and the smallest real difference (SRD) were calculated as an indication of reliability and measurement error.

## Results

Results demonstrated mean [SD] longitudinal excursion of 3.01 [0.97] mm. The between session intra-rater reliability was excellent (ICC=0.93; 95% CI, 0.70-0.96), with SEM, 0.26mm and a mean SRD of 0.75mm.

## Conclusions

Ultrasound imaging in conjunction with cross correlation analysis presents a reliable technique to quantify *in-vivo* tibial nerve movement during ankle joint dorsiflexion.
